# Wounds and the Microbiota: The Healing Interplay Between Host and Microbial Communities

**DOI:** 10.3390/ijms262311365

**Published:** 2025-11-24

**Authors:** Raghad Al-Taweel, Ayat S Hammad, Ali Tajammul, Sergio Crovella, Maha Al-Asmakh

**Affiliations:** 1 Department of Biomedical Sciences, College of Health Sciences, QU Health, Qatar University, Doha P.O. Box 2713, Qatar; 2 Biomedical Research Center, Qatar University, Doha P.O. Box 2713, Qatar

**Keywords:** wound healing, skin microbiota, chronic wounds, commensal bacteria, host–microbe interaction, biofilm

## Abstract

Chronic, non-healing wounds represent a major global health challenge, often aggravated by microbial dysbiosis and impaired host responses. Wound healing progresses through four overlapping phases—hemostasis, inflammation, proliferation, and remodeling—yet recent findings reveal that the skin microbiota actively participates in each step through immune, metabolic, and signaling mechanisms. Beneficial microorganisms such as *Staphylococcus epidermidis* and *Lactobacillus plantarum* promote tissue repair by inducing antimicrobial peptides and modulating cytokine production, whereas opportunistic pathogens (*Staphylococcus aureus*, *Pseudomonas aeruginosa*, *Enterococcus faecalis*) delay closure via biofilm formation and proteolytic activity. This review integrates current molecular insights and bibliometric trends to highlight advances and remaining challenges in understanding the wound–microbiome axis. A deeper grasp of these interactions can inform next-generation, microbiome-targeted therapies for chronic wounds.

## 1. Introduction

Wounds, whether acute or chronic, disrupt the skin’s protective and sensory functions and impose a substantial social and economic burden worldwide [1]. Under normal circumstances, acute wounds progress through a highly coordinated cascade of cellular and molecular events that culminate in tissue restoration and remodeling [2]. In contrast, chronic wounds deviate from this orderly process. According to Coerper et al. (2009), chronic wounds are defined as those that do not achieve at least a 50% reduction in wound area within four weeks of appropriate treatment [3]. Similarly, Leaper et al. (2008) described them as wounds exhibiting less than a 20–40% reduction in area after 2–4 weeks of optimal therapy or failing to achieve complete closure within six weeks of treatment [4]. These non-healing lesions are characterized by persistent inflammation, microbial colonization, and impaired re-epithelialization [2]. The global prevalence of chronic wounds—estimated at 1.5 to 2.2 cases per 1000 individuals—continues to rise, largely driven by population aging and the growing burden of chronic diseases [5,6].

The transition from an acute to a chronic wound state is multifactorial. Systemic influences such as age-related hormonal decline, diabetes mellitus, vascular insufficiency, and the use of medications including glucocorticoids, chemotherapeutic agents, and non-steroidal anti-inflammatory drugs (NSAIDs) are well-recognized contributors to impaired healing [2,7]. Local factors, including hypoxia, ischemia, and infection, further exacerbate tissue damage and delay repair [7,8]. However, traditional risk factors alone cannot fully explain the marked inter-individual variability in wound healing outcomes. This limitation has shifted attention toward the role of skin microbiota as a potential regulator of healing dynamics.

The skin hosts a diverse microbial ecosystem dominated by *Staphylococcus*, *Cutibacterium*, *Corynebacterium*, and *Streptococcus* species [9]. These commensal communities engage in continuous bidirectional communication with keratinocytes and immune cells through pattern-recognition receptors such as Toll-like and Nucleotide-binding oligomerization domain–like receptors (NOD-like receptors, NLRs), modulating inflammation, angiogenesis, and re-epithelialization. When this balance is disturbed—a state termed dysbiosis—the healing process may be disrupted. Dysbiosis, driven by factors such as aging, hormonal fluctuations, antibiotic exposure, altered moisture, or oxygen deprivation, has been linked to reduced expression of tight-junction proteins, elevated pH, and sustained inflammatory signaling, ultimately predisposing wounds to chronicity [5,10].

Recent multi-omics studies have provided compelling evidence that specific bacterial consortia correlate with distinct wound phenotypes [7,9]. For instance, microbiota dominated by *Pseudomonas* and certain *Streptococcus* groups are associated with delayed closure, while communities enriched in *Lactobacillus* and *Staphylococcus epidermidis* correspond with improved healing outcomes [11]. These findings underscore that wound repair is not a sterile or unidirectional process but rather a dynamic interplay between the host immune system and resident microorganisms.

**Figure 1:**
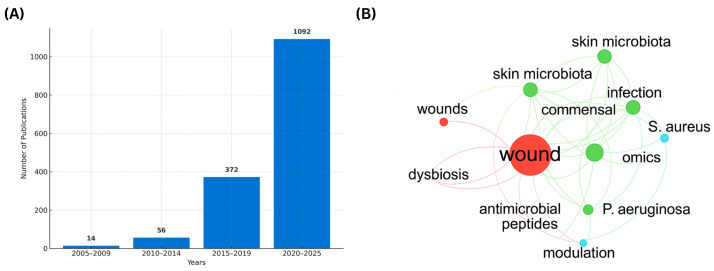
Trends and Research Clusters in Wound Microbiome Studies (2005–2025). (**A**) Temporal distribution of publications retrieved from Scopus and Web of Science using the query “wound microbiome” OR “skin microbiota AND healing.” Research output rose sharply from 14 papers in 2005–2009 to 1092 in 2020–2025. (**B**) VOSviewer version 1.6.20 for Mac, released on 31 October 2023 keyword co-occurrence map highlighting four main thematic clusters: red: chronic wound, biofilm, and dysbiosis; green: infection, skin microbiota, commensal organisms, inflammation, and diagnostic approaches; blue: probiotics, antimicrobial peptides, modulation, *S. aureus*, and *P. aeruginosa*; yellow: omics-based methodologies.

To contextualize the accelerating progress in this field, a bibliometric analysis was conducted covering the period 2005–2025 using Scopus and Web of Science databases with the query “wound microbiome” OR “skin microbiota AND healing.” A total of 1534 indexed publications were retrieved. As shown in Figure 1, the number of studies rose exponentially—from 14 papers between 2005–2009 to 1092 between 2020–2025—driven by the integration of next-generation sequencing, metagenomics, and systems-biology approaches. Keyword co-occurrence mapping revealed four major thematic clusters: (i) chronic wounds, biofilm, and dysbiosis; (ii) infection, skin microbiota, commensal immunity, and diagnostics; (iii) probiotics, antimicrobial peptides, *S. aureus*, and *P. aeruginosa*; and (iv) omics-based methodologies. Together, these trends illustrate the rapid evolution of the wound-microbiome field from descriptive culture-based studies to multi-omics and translational frameworks linking microbial ecology with host immune and metabolic regulation.

Accordingly, this review aims to (i) elucidate the mechanistic role of microbiota to the sequential phases of wound healing, (ii) synthesize emerging diagnostic and therapeutic strategies, and (iii) outline future directions in molecular wound microbiology.

## 2. Anatomy and Physiology of the Skin

**Figure 2:**
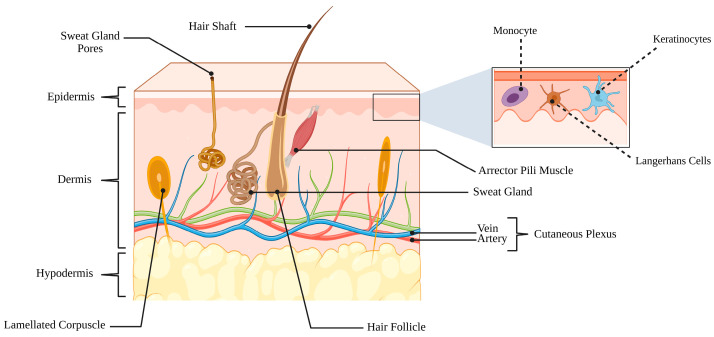
Schematic representation of the three principal layers of skin. The outermost epidermis provides a protective barrier and contains specialized cells, including keratinocytes, melanocytes, and Langerhans cells. Beneath it, the dermis comprises connective tissue, hair follicles, sebaceous glands, sweat glands, blood vessels, and sensory nerve endings, supporting both structural integrity and physiological function. The deepest layer, the hypodermis (subcutaneous tissue), is primarily composed of adipose tissue and serves as insulation, energy storage, and cushioning. (Created in BioRender. Hammad, A. (2025) https://BioRender.com/w8ealse).

The skin is a complex, multifunctional organ that constitutes the body’s first line of defense against environmental, chemical, and microbial stressors. It acts not only as a mechanical and chemical barrier but also as an immunologically active and metabolically active tissue that constantly interacts with its resident microbiota [12,13]. Structurally, the skin is composed of three major layers—the epidermis, dermis, and hypodermis—whose coordinated organization ensures protection, thermoregulation, immune surveillance, and wound repair Figure 2 [12].

### 2.1. The Epidermis

The epidermis is a stratified squamous epithelium composed mainly of keratinocytes, which synthesize keratin—a structural protein that maintains barrier integrity—and secrete cytokines and antimicrobial peptides in response to injury [14,15]. The layer is organized into distinct strata, from the stratum basale, which contains mitotically active basal cells, to the stratum corneum, composed of flattened, keratin-filled corneocytes embedded in a lipid-rich matrix that limits transepidermal water loss and protects against external insults [14,15]. Intermediate layers—the stratum spinosum, stratum granulosum, and, in acral skin, the stratum lucidum—collectively coordinate keratinocyte differentiation, keratin filament aggregation, and the formation of protein–lipid envelopes [16]. Together, these layers mediate the process of keratinization and sustain the epidermal permeability barrier through enzymatic cross-linking of structural proteins and lipid organization [16].

In addition to its physical barrier role, the epidermis functions as an immune and microbial interface. Keratinocytes express pattern-recognition receptors (PRRs), including Toll-like receptor 2 (TLR2), Toll-like receptor 4 (TLR4), and C-type lectin receptors that detect microbial-associated molecular patterns (MAMPs) and activate NF-κB–dependent transcription of cytokines such as IL-1β and TNF-α [17,18,19,20,21]. This signaling leads to the release of chemokines and antimicrobial peptides (AMPs), including defensins, cathelicidin (LL-37), and S100 proteins, which exhibit both direct antibacterial activity and chemoattractant functions for immune cells [19,20]. Keratinocytes also communicate with Langerhans cells and dermal dendritic cells through cytokines and extracellular vesicles, bridging innate and adaptive immunity [19,22].

Commensal bacteria such as *Staphylococcus epidermidis* reinforce this defense by stimulating β-defensin and LL-37 expression via TLR2 signaling, thereby maintaining immune homeostasis and barrier function [18,23,24,25]. Conversely, disruptions in lipid composition, pH, or oxygen levels impair these interactions, predisposing the epidermis to infection and delayed wound repair [26].

### 2.2. The Dermis

The dermis lies beneath the epidermis and provides mechanical strength, elasticity, and vascular support [13]. It consists predominantly of collagen and elastin fibers synthesized by fibroblasts and embedded in a proteoglycan-rich extracellular matrix that maintains hydration and resilience [15]. This layer is richly vascularized and innervated, housing blood vessels, lymphatics, and sensory nerve endings essential for nutrient exchange, thermoregulation, and rapid immune responses [12].

Physiologically, fibroblasts, endothelial cells, and macrophages orchestrate repair through the release of growth factors such as Vascular Endothelial Growth Factor (VEGF) and Fibroblast Growth Factor 2 (FGF2), promoting angiogenesis and collagen deposition [27,28,29,30,31]. Emerging evidence suggests that microbial metabolites, particularly short-chain fatty acids (SCFAs) such as acetate, propionate, and butyrate, may modulate dermal fibroblast proliferation, collagen synthesis, and endothelial activity through pathways analogous to GPR41/43-mediated signaling described in intestinal and vascular systems [32]. In the skin, these metabolites are proposed to exert anti-inflammatory and vasoregulatory effects by attenuating cytokine release and adhesion-molecule expression via MAPK modulation and histone deacetylase inhibition, thereby contributing to the preservation of vascular and immune homeostasis [32].

Dermal macrophages integrate immune and microbial cues, polarizing toward M1-like (pro-inflammatory) or M2-like (reparative) phenotypes [14,33]. M1 macrophages secrete TNF-α, IL-1, and IL-6, initiating inflammation, whereas M2 subsets release VEGF, FGF2, CCL2, and IGF-1, driving angiogenesis and granulation-tissue formation [27,28,29,30,31]. Under hypoxia, hypoxia-inducible factor-1α (HIF-1α) stabilization induces VEGF-A, CXCR4, SDF-1α, and TGF-β expression, coordinating cellular metabolism and tissue remodeling [34].

### 2.3. The Hypodermis

The hypodermis, or subcutaneous layer, consists primarily of adipocytes, fibroblasts, and macrophages, embedded in a loose connective-tissue matrix [13]. It provides mechanical cushioning, insulation, and energy storage, while contributing to vascular and immune regulation [35]. Adipocytes in the hypodermis have been shown to express Toll-like receptors (notably TLR2 and TLR4) and to respond to microbial ligands via TLR4 up-regulation of TLR2 [36,37]. Adipocyte-derived signals such as leptin also modulate pro-inflammatory responses [37]. While these data suggest adipocytes may contribute to innate immune activation (including NF-κB signalling) and potentially antimicrobial peptide production in skin [36,37], direct demonstration of hypodermal adipocyte-derived LL-37 (cathelicidin) in wound-healing contexts remains to be established.

During hypoxia, HIF-1α activation stimulates VEGF-mediated angiogenesis, generating oxygen gradients that modulate both immune-cell recruitment and microbial colonization dynamics at the wound interface [38,39]. HIF-1α also governs the transcription of multiple chemokines and growth factors that facilitate tissue repair and regeneration [40,41].

### 2.4. Functional Integration

Collectively, the epidermis, dermis, and hypodermis operate as a structurally and functionally integrated system that combines barrier protection, immune regulation, thermoregulation, and microbial symbiosis. The skin’s anatomical complexity is tightly linked to its physiological adaptability, enabling it to respond dynamically to injury and infection. Disruption of this equilibrium—by ischemia, infection, or dysbiosis—compromises healing and fosters chronic inflammation. Understanding this structural–functional interdependence provides the biological foundation for elucidating how the skin microbiota modulates each phase of wound repair.

Beyond this structural and functional crosstalk, each cutaneous compartment harbors distinct microbial communities that occupy defined spatial niches and contribute to barrier maintenance and repair, as demonstrated by Gallo and colleagues [42]. In healthy skin, the superficial epidermis is dominated by *S. epidermidis*, one of the most abundant commensal species at this level [43]. Across epidermal and dermal layers, microbial DNA is composed predominantly of *Proteobacteria*, with comparatively lower contributions from *Actinobacteria*, *Firmicutes* and *Bacteroidetes* [44]. *Lactobacillus* spp. further support epidermal hydration by producing lactate, which promotes ceramide synthesis within the stratum corneum [45]. Regional differences refine these patterns: sebaceous sites such as the forehead and upper back are enriched in lipophilic commensals including *Cutibacterium acnes*, which metabolizes sebum into fatty acids and thereby sustains barrier integrity [46], whereas moist intertriginous areas favor *Staphylococcus* and *Corynebacterium* species that adapt to humid microenvironments; notably, *Corynebacterium* also constitutes a dominant genus in several dry habitats [47,48]. Collectively, these observations indicate that *Proteobacteria* and associated commensals colonize both superficial and deeper cutaneous compartments and contribute to skin homeostasis at the interface between host tissues and the external environment [49].

When the barrier is disrupted, these resident taxa overlap with those recovered from pathological settings: in surgical site infections and chronic wounds, *Staphylococcus* spp. are ubiquitous, while *C. acnes* and *P. aeruginosa* are also commonly isolated despite their presence on clinically healthy skin [50,51]. Along the vertical axis of the stratum corneum, oxygen gradients further structure these communities, with aerobic *Corynebacteriaceae* and anaerobic *Peptostreptococcales* exhibiting depth-dependent shifts in relative abundance [52]. In addition, cutaneous appendages—including hair follicles, sebaceous glands and eccrine ducts—serve as specialized microenvironments enriched in *C. acnes*, *coagulase-negative staphylococci* and *Corynebacterium* spp., which act as microbial reservoirs capable of reseeding the surface and modulating local immune responses after injury [50,51]. At the level of the hypodermis, dermal white adipose tissue functions as an immunologically active compartment: dermal adipocytes proliferate and upregulate the antimicrobial peptide cathelicidin in response to *S. aureus* skin infection, sensing microbial products through pattern-recognition receptors and thereby linking deep bacterial cues to cytokine and antimicrobial-peptide production in the cutaneous defense network [53,54]. Together, these data highlight that microorganisms distributed across the epidermis, appendages, dermis and hypodermis not only occupy distinct anatomical niches but also influence barrier integrity and the trajectory of wound healing when homeostasis is disturbed.

## 3. Phases of Wound Healing: Cellular Dynamics and Microbiome Modulation [50,51]

**Figure 3:**
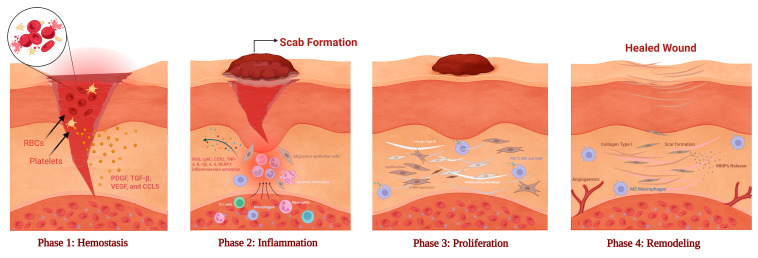
Sequential phases of wound healing. Wound healing proceeds through four overlapping phases. (**Phase 1**) Hemostasis: Platelets aggregate with red blood cells (RBCs) and release growth factors (PDGF, TGF-β, VEGF, CCL5) to initiate clotting and recruit immune cells. (**Phase 2**) Inflammation: Neutrophils and macrophages infiltrate the wound, releasing ROS and cytokines (TNF-α, IL-1β, IL-6) to clear pathogens and trigger repair. (**Phase 3**) Proliferation: Fibroblasts deposit type III collagen and form granulation tissue, while angiogenic factors (FGF-2, EGF, VEGF) promote neovascularization and epithelial migration. (**Phase 4**) Remodeling: Type III collagen is replaced by type I collagen, leading to extracellular-matrix reorganization, scar formation, and restored tensile strength. M2 macrophages and MMPs coordinate collagen turnover and vascular maturation. (Created in BioRender. Hammad, A. (2025) https://BioRender.com/w9h4io6).

Wound healing is an evolutionarily conserved, multistage process involving four partially overlapping yet distinct phases—hemostasis, inflammation, proliferation, and remodeling—each orchestrated by intricate signaling between immune, stromal, and microbial factors Figure 3 [35]. Wound repair is now recognized as a dynamic host–microbe interaction, where commensal and pathogenic species modulate inflammation, angiogenesis, and tissue regeneration [11,55].

### 3.1. Hemostasis: Platelet Activation and Microbial Sensing

The hemostatic phase begins within seconds after injury, arresting bleeding and initiating tissue defense [56]. Platelets adhere to exposed subendothelial collagen via von Willebrand factor and glycoprotein receptors, forming a fibrin-rich clot that seals the wound and provides a provisional scaffold for migrating cells [57]. Upon activation, platelets degranulate, releasing growth factors (e.g., PDGF, TGF-β, VEGF) and chemokines (e.g., CCL5), together with thrombin, which recruit fibroblasts and endothelial cells and promote early angiogenesis [58,59,60].

Beyond coagulation, platelets and keratinocytes contribute to innate immune sensing in the early wound milieu. Keratinocytes express PRRs, including TLR2, and respond to staphylococcal lipoteichoic acid (LTA); *in vitro* studies further show that *Staphylococcus epidermidis* can activate aryl hydrocarbon receptor (AhR) signaling in keratinocytes, increasing IL-1α/IL-1β and β-defensin-3 expression [61]. Platelets also express TLR2 and can be activated by Gram-positive ligands such as LTA [61], suggesting potential cross-talk with epidermal defenses; however, a direct platelet-TLR2 → keratinocyte-AhR cascade in human skin wounds has not yet been demonstrated and remains an area for future investigation. In contrast, TLR4 engagement by lipopolysaccharide (LPS), as in *P. aeruginosa* exposure, can amplify NF-κB–dependent inflammation, potentially destabilizing early clot structure and impairing repair [62]. Thus, even the initial wound microenvironment is microbially reactive, with commensal cues likely fine-tuning inflammation while pathogen-associated signals can derail orderly healing.

### 3.2. Inflammation: Cytokine Regulation and Biofilm-Mediated Persistence

The inflammatory phase (6–72 h) recruits neutrophils and monocytes that eliminate pathogens and necrotic tissue by releasing proteolytic enzymes and reactive oxygen species (ROS) [63,64,65]. Controlled ROS production aids microbial clearance, but overproduction oxidizes the extracellular matrix (ECM) and prolongs tissue injury [66]. As neutrophils undergo apoptosis, monocytes differentiate into M1 macrophages, expressing Ly6C and CCR2, which mediate phagocytosis of debris and apoptotic neutrophils, marking the transition to tissue repair [56,67,68]. Activated M1 macrophages release TNF-α, IL-1β, IL-6, and cyclooxygenase-2, sustaining defense mechanisms [67], while neutrophil extracellular traps (NETs) and NOD-like receptor protein 3 (NLRP3) inflammasome activation further amplify IL-1β release through TLR4/TLR9/NF-κB signaling [69,70].

Commensal bacteria promote the resolution of this phase. *S. epidermidis* phenol-soluble modulins induce IL-10 and TGF-β, reducing excessive inflammation [71]. Conversely, pathogenic species such as *P. aeruginosa* sustain chronic inflammation via quorum-sensing systems (las, rhl) that coordinate biofilm formation and the release of elastases and rhamnolipids [72,73]. Elastase can degrade thrombin, generating peptides such as FYT21 that block TLR dimerization and suppress innate signaling [74]. Biofilms establish hypoxic microdomains, stabilizing HIF-1α, which paradoxically maintains VEGF production but impairs macrophage polarization and neutrophil function, thereby delaying closure [75,76]. Consequently, persistent biofilm activity sustains a non-resolving inflammatory state characteristic of chronic wounds.

### 3.3. Proliferation: Fibroblast Activation and Microbial-Derived Mediators

The proliferative phase (days 3–10) is characterized by fibroblast proliferation, angiogenesis, and re-epithelialization [77]. Fibroblasts secrete matrix metalloproteinases (MMPs) to degrade the provisional fibrin clot [78], while depositing fibronectin, hyaluronic acid, proteoglycans, and type III collagen to establish a new extracellular scaffold [79]. Concurrently, angiogenic growth factors such as FGF-2, Epidermal growth factor (EGF), and VEGF stimulate vascular sprouting to restore oxygen and nutrient supply [11,80].

Fibroblasts differentiate into myofibroblasts, expressing α-smooth muscle actin (α-SMA) through TGF-β-dependent signaling, which promotes contraction and tensile-strength restoration [57,81,82,83]. Microbial metabolites, particularly short-chain fatty acids (acetate, propionate, butyrate), enhance fibroblast migration and collagen synthesis through GPR41/43 signaling and histone-deacetylase inhibition, reinforcing ECM deposition [84,85]. This reciprocal metabolic cross-talk between host and microbiota may influence the trajectory of repair vs chronicity.

### 3.4. Remodeling: ECM Turnover and Microbial Resolution

The remodeling phase entails replacement of type III collagen with type I, increased cross-linking, and granulation-tissue reorganization [77]. Myofibroblasts, regulated by TGF-β and mechanical stress, drive ECM synthesis, MMP release, and wound contraction [86,87,88]. Transcriptomic analyses identify COL4A1, COL4A2, and COL6A1 as hub genes mediating ECM remodeling and protein-receptor interactions [89]. Genes encoding TGF-β1 and MMPs are upregulated to balance collagen deposition and ECM resorption [90]. Dysregulated cytokine or MMP expression disrupts this balance, predisposing to fibrotic scarring or chronic non-healing wounds [90,91].

The microbiome continues to influence this stage indirectly. *Staphylococcus epidermidis* can augment antimicrobial-peptide programs, including β-defensin expression [92,93,94]. *Corynebacterium* taxa interact with lipid niches of the skin and have been linked to shifts in barrier lipids that may secondarily influence extracellular-matrix organization [92]. Likewise, *Cutibacterium acnes* shapes inflammatory and matrix-remodeling pathways—engaging TLR2/TLR4 signaling and promoting MMP expression, partly via secreted short-chain fatty acids—principally in in vitro and preclinical models [95,96]. By contrast, *P. aeruginosa* proteases, notably pseudolysin (LasB) and protease IV, degrade collagen, blunt neovascularization, and delay re-epithelialization [97]. Collectively, the wound microbiome can fine-tune barrier defense and matrix dynamics through commensal cues, whereas pathogen-derived factors tend to disrupt these processes.

Emerging multi-omics studies indicate that successful wound resolution is marked by restoration of commensal microbial dominance [11], underscoring microbiome re-equilibration as a molecular hallmark of healing.

## 4. Chronic Wound Healing and the Skin Microbiome

### 4.1. Chronic Wound Healing: Pathophysiological Context

A chronic wound is defined as a cutaneous lesion that fails to progress through the orderly stages of healing or does not achieve closure within twelve weeks of appropriate treatment [98]. Its persistence reflects a multifactorial disruption of the repair cascade, where intrinsic and extrinsic factors—including malnutrition, tissue hypoxia, microbial colonization, immunosuppression, chronic diseases, and genetic predispositions—converge to impair tissue regeneration [98].

Distinct microbial and immunological dynamics differentiate acute from chronic wound infections. Acute wounds are dominated by metabolically active, planktonic microorganisms that trigger a robust inflammatory response through virulence-factor expression, promoting pathogen clearance and repair initiation. In contrast, chronic wounds harbor sessile, biofilm-forming microbial consortia that exist in a metabolically quiescent state, shielded within extracellular polymeric matrices [99,100]. These biofilms blunt host immune activation, leading to a dysregulated, low-grade inflammatory milieu dominated by persistent neutrophil infiltration and ineffective phagocytosis [41,56]. As a result, neutrophils release proteolytic enzymes and ROS that exacerbate oxidative stress, cause collateral tissue injury, and induce fibroblast and keratinocyte senescence [41,57].

A defining feature of chronic wounds is sustained inflammation driven by an imbalance between proteolytic enzymes and their inhibitors. Elevated MMP activity—particularly MMP-2 and MMP-9—alongside reduced tissue inhibitor of metalloproteinase (TIMP) expression, leads to excessive ECM degradation and impaired granulation-tissue formation [101,102,103,104,105,106]. Persistent stimulation by pro-inflammatory cytokines such as IL-1β and TNF-α maintains macrophages and fibroblasts in a pro-inflammatory state, preventing their transition to reparative phenotypes [107,108,109,110]. This inflammatory microenvironment amplifies oxidative stress, resulting in DNA damage, mitochondrial dysfunction, and cell-cycle arrest—hallmarks of cellular senescence that further suppress epithelial proliferation and tissue remodeling [106,107,111,112,113,114,115].

Hypoxia is another critical factor impeding chronic wound healing. In the early stages of healing, the high metabolic demand of infiltrating cells rapidly depletes available oxygen, creating a hypoxic microenvironment [7]. Prolonged hypoxia disrupts the cellular and molecular processes necessary for effective healing [116,117]. Concurrently, infection complicates the wound milieu, as microbial access to deeper tissues following skin injury introduces further challenges. The replication status of these invading microorganisms—particularly the shift to biofilm formation—significantly influences infection severity and persistence [7].

Beyond local wound conditions, systemic influences profoundly affect healing outcomes. Advanced age, diabetes mellitus, obesity, malnutrition, psychological stress, smoking, and excessive alcohol consumption all compromise immune competence and tissue-repair capacity [7]. Additionally, pharmacological agents such as glucocorticoids and chemotherapeutic drugs suppress cell proliferation, angiogenesis, and inflammatory resolution, further delaying wound closure [7]. Together, these systemic and lifestyle factors create a physiological environment that hinders effective regeneration and predisposes to chronic, non-healing wounds.

### 4.2. The Skin and Wound Microbiome Composition

The human skin harbors a diverse and dynamic microbial ecosystem comprising bacteria, fungi, viruses, and archaea that collectively contribute to immune regulation, barrier integrity, and tissue homeostasis [118]. Far from passive colonizers, these microorganisms act as metabolic and immunological partners, dynamically responding to environmental cues such as moisture, pH, and oxygen gradients. Taxonomic and metagenomic analyses reveal that microbial composition varies across body sites, reflecting distinct microenvironments and host–microbe interactions [119].

#### 4.2.1. Commensal Communities and Protective Roles

In healthy skin, the dominant bacterial phyla include Actinobacteria (e.g., *Cutibacterium*, *Corynebacterium*), Firmicutes (e.g., *Staphylococcus*, *Streptococcus*), Proteobacteria, and Bacteroidetes [48]. Among these, *Staphylococcus epidermidis* is a keystone commensal that plays a pivotal protective role. It secretes AMPs such as β-defensins and LL-37, the lipopeptide LP78, and the serine protease inhibitor Esp, which collectively activate TLR2 signaling and stimulate LTA production [1,19,120]. This signaling cascade suppresses TLR3-mediated inflammation and promotes epithelial repair. Ex vivo studies reveal that epidermal keratinocytes and Langerhans cells can discriminate between commensal *S. epidermidis* and pathogenic *S. aureus*: the former induces minimal IL-1β secretion, preserving immune tolerance, whereas the latter triggers robust pro-inflammatory signaling [121].

Specific *S. epidermidis* strains expressing glutamyl endopeptidase (Esp) inhibit *S. aureus* biofilm formation by degrading its surface adhesion proteins [104,105]. The combined action of Esp and host β-defensins exhibits potent bactericidal activity against *S. aureus* within biofilms [122]. Moreover, through TLR2–EGFR cross-talk, *S. epidermidis* induces TGF-α–mediated β-defensin-3 (hBD3) expression, reinforcing cutaneous innate immunity [92,123]. 

Similarly, *Cutibacterium acnes* contributes to immune homeostasis by producing SCFAs and activating the TLR2/IL-6/STAT3 pathway, which regulates MMP-9 expression and influences keratinocyte differentiation [124]. Together, these commensals maintain a balanced inflammatory tone that supports barrier function and epithelial renewal.

#### 4.2.2. Dysbiosis and Pathogenic Shifts

In the wound environment, barrier disruption, oxygen depletion, and altered nutrient gradients create a niche favorable to facultative and obligate anaerobes [125]. Chronic wounds exhibit reduced microbial diversity but increased dominance of pathogenic taxa such as *Staphylococcus aureus*, *Pseudomonas aeruginosa*, *Enterococcus faecalis*, and *Proteus mirabilis* [126,127]. Gram-positive pathogens express microbial surface components recognizing adhesive matrix molecules (MSCRAMMs) that mediate tissue adhesion and invasion [128].

These pathogens form polymicrobial biofilms, facilitating immune evasion and antibiotic tolerance. Quorum-sensing systems—agr in *S. aureus* and las/rhl in *P. aeruginosa*—regulate virulence gene expression, extracellular polymeric substance (EPS) synthesis, and phenotypic resistance [129,130,131]. Efflux pumps such as MexAB–OprM and AcrAB–TolC further enhance multidrug resistance and promote biofilm resilience [99,132]. Metatranscriptomic profiling demonstrates that chronic wound microbiota display sustained transcription of virulence pathways (siderophore biosynthesis, protease secretion) despite antimicrobial exposure [100].

#### 4.2.3. Functional Interplay and Spatial Zonation

Advances in spatially resolved sequencing and high-resolution imaging have revealed striking heterogeneity within the wound microbiome, characterized by vertical and metabolic gradients across wound depths. Surface layers are dominated by aerobic species, while deeper, oxygen-depleted niches harbor facultative and obligate anaerobes, including *Pseudomonas*, *Finegoldia*, and *Anaerococcus* [133]. This spatial stratification mirrors metabolic heterogeneity—encompassing gradients of oxygen, lactate, and ROS which collectively shape microbial composition and host immune activation.

Among these factors, ROS play a dual role as both antimicrobial and signaling mediators. The quorum-sensing regulator LasR in *P. aeruginosa* is highly sensitive to oxidative stress; mutations in LasR increase susceptibility to nitrosative damage due to endogenous nitric oxide (NO) accumulation, compromising bacterial survival under high-cell-density conditions [134]. Conversely, *P. aeruginosa* counteracts oxidative injury through antioxidant systems and NO-detoxifying enzymes, which enhance persistence and attenuate host inflammatory responses [134]. This delicate balance allows pathogenic species to withstand immune stress while maintaining chronic infection.

Under hypoxic conditions, pathogen-derived metabolites further reprogram host immune metabolism. Neutrophils shift toward aerobic glycolysis (Warburg-like metabolism), generating elevated lactate via NADPH oxidase (NOX)– and HIF-1α–dependent mechanisms [135]. Accumulated lactate signals through endothelial GPR81 receptors, downregulating VE-cadherin and increasing vascular permeability to facilitate leukocyte infiltration [135]. Elevated lactate levels also modulate macrophage polarization, promoting an M2-like, pro-repair but immunosuppressive phenotype characterized by upregulation of ARG1, VEGFA, IL-10, and TGFB1 [136,137,138,139,140]. Although this phenotype enhances angiogenesis and ECM remodeling, it concurrently dampens antimicrobial activity, prolonging inflammation and contributing to delayed wound resolution [141].

Beyond HIF-dependent responses, recent evidence identifies a HIF-independent pseudo-hypoxic pathway mediated by N-Myc downstream-regulated gene 3 (NDRG3) [141]. Normally degraded through PHD2–VHL pathways, NDRG3 binds to lactate under hypoxia, preventing degradation and activating the Raf–ERK signaling cascade [141,142]. This promotes angiogenesis and fibroblast proliferation but, if unresolved, sustains aberrant remodeling and fibrosis. Thus, hypoxia and lactate metabolism cooperatively orchestrate both HIF-dependent and -independent mechanisms, integrating microbial cues with host metabolism to shape immune recruitment, angiogenesis, and tissue repair dynamics [141].

Recent single-cell RNA sequencing (scRNA-seq) studies further demonstrate that spatial microbial architecture directly dictates host cell phenotypes [143]. Fibroblasts adjacent to *Pseudomonas* biofilms exhibit a senescence-associated transcriptional profile, expressing MMP-9 and downregulating collagen synthesis [143]. Conversely, fibroblasts near commensal-dominant zones upregulate antioxidant and pro-repair genes, including SOD2 and COL1A1 [101]. Collectively, these findings reveal that microbial spatial organization is a critical determinant of cellular fate, matrix remodeling, and wound chronicity, providing a mechanistic link between microbial ecology and host tissue behavior.

### 4.3. Virome and Mycobiome Contributions

Although bacteria constitute the majority of the wound microbiota, the virome and mycobiome—comprising bacteriophages and fungi—play substantial regulatory roles in modulating microbial ecology and host immune responses [102].

Bacteriophages exert context-dependent immunomodulatory effects. They can attenuate inflammation driven by bacterial LPS while eliciting only mild cytokine responses when present alone [144]. Mechanistically, phages suppress TLR4 and upregulate TLR10, leading to reduced expression of pro-inflammatory cytokines (TNF-α, IL-6, IL-8, CXCL5) and enhanced production of anti-inflammatory mediators (IL-10, SOCS-3, IL-1RN) [145]. In experimental models, the *P. aeruginosa* phage Pf4 decreased LPS-induced TNF-α, IL-1α, IL-1β, IL-6, CXCL1, CXCL5, and GM-CSF secretion in human macrophages, and limited neutrophil recruitment in murine wounds through TLR3–IFNAR–dependent pathways, highlighting the species-specific immunoregulatory role of phages [146]. Additionally, phages targeting *Staphylococcus aureus* and *P. aeruginosa* can disrupt biofilm architecture and modulate microbial population dynamics, underscoring their potential as therapeutic microbiome modulators [103].

Fungal communities, though less abundant, also exert important effects on wound immunity and healing. Common taxa, such as *Candida albicans* and *Malassezia restricta*, activate the Dectin-1 and TLR2 signaling pathways [104]. TLR2 recognizes fungal lipoproteins and zymosan, signaling via MyD88 to activate NF-κB and MAPK cascades (ERK, JNK, p38) [147]. Functional cooperation between Dectin-1 and TLR2 amplifies pro-inflammatory cytokine production (IL-1β, TNF-α, IL-6) and enhances phagocytic responses [147].

Collectively, these findings illustrate that bacteriophages and fungi exert mechanistic control over host immunity, bacterial behavior, and biofilm dynamics. Nevertheless, the molecular crosstalk among bacterial, viral, and fungal consortia in wounds remains poorly defined and represents an emerging frontier in wound microbiology. Thus, the integration of bacteriophage and fungal signaling into wound-microbiome models will be essential to fully capture the polymicrobial ecology of chronic wounds.

## 5. Molecular Mechanisms of Microbiota-Mediated Wound Modulation

The molecular dialogue between the host and its resident microbiota orchestrates every phase of cutaneous wound healing—from inflammation to tissue remodeling. This communication relies on a sophisticated network of PRRs, quorum-sensing (QS) signals, AMPs, redox modulators, and metabolic reprogramming pathways. Whether a wound progresses toward resolution or chronicity is determined by the balance of these interactions, which can be disrupted under conditions such as hypoxia, oxidative stress, or dysbiosis.

### 5.1. Pattern-Recognition Receptor Signaling

The first layer of host–microbe communication occurs through PRRs, including Toll-like receptors (TLRs) and NLRs, expressed by keratinocytes, macrophages, and dendritic cells [105]. These receptors detect MAMPs and translate them into immune and reparative cues.

Commensal species such as *Staphylococcus epidermidis* provide regulatory ligands—most notably LTA—that activate TLR2 signaling [106]. This controlled activation induces antimicrobial peptides (β-defensins, LL-37) via the MyD88–NF-κB–MAPK axis (ERK, JNK, p38), enhancing barrier integrity without provoking destructive inflammation [106,148]. In contrast, *P. aeruginosa* LPS hyperactivates TLR4, leading to sustained NF-κB signaling, overproduction of IL-1β, IL-6, and TNF-α, and chronic neutrophil infiltration [107,149]. Recent experimental evidence supports this dichotomy: exposure of dermal fibroblasts to virulence-factor secretomes from pathogenic bacteria induced a pro-inflammatory cytokine profile characterized by elevated IL-6 and IL-8 and reduced TGF-β and VEGF, whereas commensal-derived preparations—such as those from *Lactobacillus plantarum*—produced the opposite pattern, consistent with TLR2-mediated homeostatic signaling [150].

Importantly, TLR2 also licenses NLRP3 inflammasome activation, providing the priming signal for caspase-1–dependent maturation of IL-1β and IL-18 [151]. Thus, TLR2 maintains a delicate equilibrium—promoting inflammation necessary for repair while preventing uncontrolled tissue injury. The reciprocal regulation between TLR2 and TLR4 represents a molecular switch that determines whether microbial recognition culminates in regeneration or chronic inflammation.

### 5.2. Quorum Sensing and Biofilm-Driven Pathogenicity

QS is a population density–dependent communication mechanism utilized by both Gram-positive and Gram-negative bacteria to coordinate group behaviors essential for survival and pathogenicity [152]. This process regulates a wide array of physiological activities—including virulence expression, genetic competence, secondary metabolite synthesis, motility, and biofilm formation—allowing bacteria to function as multicellular-like communities [152].

Mechanistically, QS relies on the production, secretion, and detection of small diffusible signaling molecules termed autoinducers (AIs) [72]. Gram-negative bacteria predominantly use acyl-homoserine lactones (AHLs), whereas Gram-positive species rely on processed oligopeptides, known as autoinducing peptides (AIPs), to modulate virulence and biofilm maturation [72]. By sensing extracellular AI concentrations, bacterial populations synchronize gene expression to optimize environmental adaptation and host colonization.

In *Staphylococcus aureus*, the accessory gene regulator (agr) system represents the archetype of QS-mediated virulence control [72]. The agr circuit modulates biofilm formation and toxin production in a cell-density–dependent manner. At low cell density (LCD), agr signaling remains inactive, favoring the expression of surface adhesins that promote attachment and initial biofilm establishment [72]. As bacterial density increases, accumulating AIPs—synthesized from AgrD and processed and exported by AgrB—bind and activate the AgrC/AgrA two-component system. Phosphorylated AgrA subsequently stimulates the P2 and P3 promoters, leading to transcription of RNAII (agr operon) and RNAIII, which post-transcriptionally enhance α-toxin, protease, and hemolysin expression while repressing the rot (repressor of toxins) regulator [72,153,154]. Consistent with these regulatory outcomes, *in vitro* exposure of dermal fibroblasts to S. aureus virulence-factor preparations markedly reduced cell viability and delayed scratch-wound closure, confirming the cytotoxic and wound-impairing potential effects of *agr*-controlled secreted factors [150]. This molecular switch effectively downregulates adhesion, disperses biofilms, and induces virulence, facilitating immune evasion and tissue invasion.

In *Pseudomonas aeruginosa*, QS regulation is orchestrated through the Las and Rhl signaling hierarchies [72]. The LasI/LasR system synthesizes and detects 3-oxo-C12-homoserine lactone (3OC12-HSL), while the RhlI/RhlR circuit uses C4-HSL as its signaling molecule [72]. At high population density, LasR–3OC12-HSL complexes activate genes encoding major virulence determinants—including elastases, proteases, rhamnolipids, and exotoxin A—as well as enzymes that promote biofilm maturation [73,155,156,157]. The resulting biofilms act as physical and biochemical barriers, impairing fibroblast migration, inhibiting collagen deposition, and delaying re-epithelialization, thereby transforming acute wounds into chronic, recalcitrant lesions.

Given their central role in biofilm resilience and immune evasion, QS systems have emerged as attractive therapeutic targets. Strategies such as QS inhibition (QSI) using small-molecule antagonists, synthetic peptide mimetics, or phage-encoded quorum-quenching enzymes have demonstrated efficacy in disrupting biofilm integrity, reducing virulence, and accelerating wound closure in experimental models [158]. Such interventions hold promise for the development of next-generation, microbiome-informed treatments for chronic wound infections.

### 5.3. Antimicrobial Peptides and Host–Microbe Equilibrium

AMPs such as LL-37, human β-defensins (hBD-1–3), and dermcidin function as dual-effectors—serving both microbicidal and immunomodulatory roles within the skin barrier [19]. Their expression is tightly regulated by both commensal-derived cues and cytokine-mediated immune signaling. In particular, *Staphylococcus epidermidis*–derived LTA activates TLR2, inducing AMP expression and fortifying the cutaneous defense network without triggering deleterious inflammation [19].

Beyond microbial stimuli, IL-17A and IL-22, produced by Th17 and innate lymphoid cells, act as potent epithelial inducers of AMPs [19]. IL-17A signals via heterodimeric IL-17 receptor complexes (IL-17RA/C and IL-17RA/D), enhancing IL-23 production and AMP gene transcription in keratinocytes [159,160]. Upon skin injury, keratinocytes upregulate IL-17RA, sensitizing the tissue to this pathway [159,160]. In parallel, IL-22 engages the IL-22R/IL-10Rβ2 receptor complex to activate STAT3, driving keratinocyte proliferation, barrier reinforcement, and epithelial regeneration [159].

Among AMPs, LL-37 (human cathelicidin) exemplifies functional versatility. Its inhibition of expression delays re-epithelialization, whereas its adenoviral overexpression markedly accelerates wound closure in murine models [161,162]. Furthermore, LL-37 amplifies TLR3 signaling by forming complexes with double-stranded RNA analog poly(I:C), reinforcing antiviral defense and pro-repair responses [20].

Maintaining AMP equilibrium is critical to wound homeostasis. Protease-mediated degradation of AMPs in chronic wounds diminishes microbial defense, while overexpression can induce cytotoxicity—both outcomes impairing healing [163,164]. Thus, the AMP–microbiome axis functions as a central regulatory node ensuring balanced inflammatory resolution and repair dynamics.

Recent advances have identified bioactive AMP analogs with enhanced wound-healing potential, including Temporins A, Tylotoin, Cathelicidin-OA1, and Cathelicidin-DM [165]. Of these, Cathelicidin-DM exhibits broad-spectrum antimicrobial activity and accelerates the repair of both infected and non-infected wounds. Mechanistically, Cathelicidin-DM activates the MAPK cascade, upregulating JNK, ERK, and p38 phosphorylation, thereby promoting fibroblast proliferation, keratinocyte migration, and collagen synthesis [165]. These findings position AMP analogs as promising molecular templates for next-generation microbiome-informed wound therapeutics.

### 5.4. Reactive Oxygen Species (ROS) and Redox Signaling

Reactive oxygen species (ROS) serve as pivotal regulators across all stages of wound healing—governing angiogenesis, cell proliferation, and tissue remodeling [166]. Controlled ROS generation, primarily via NOX and mitochondrial respiratory complexes, facilitates growth factor activation and host microbial defense [166]. However, excessive ROS or inadequate detoxification disrupts ECM integrity, oxidizes lipids and proteins, and perpetuates inflammatory loops, leading to chronic non-healing wounds [166].

Pathologically elevated ROS activate transcription factors NF-κB, AP-1, and MAPKs, stimulating MMP expression in fibroblasts and driving ECM degradation [166,167,168,169]. To counterbalance oxidative injury, the Nrf2 (nuclear factor erythroid 2–related factor 2) pathway serves as the master regulator of the antioxidant response [170,171]. Upon activation, Nrf2 translocate to the nucleus, binds antioxidant response elements (AREs), and induces genes encoding heme oxygenase-1 (HO-1), glutathione peroxidase, and superoxide dismutase, restoring redox equilibrium and protecting keratinocytes from oxidative stress [170,171].

Importantly, commensal microorganisms also participate in redox homeostasis. *Lactobacillus* spp. and *Enterococcus faecalis* synthesize or import glutathione (GSH), the primary intracellular thiol antioxidant responsible for detoxifying peroxides and reactive aldehydes, thereby supporting both microbial and host redox stability [172,173]. Topical application of *Lactobacillus delbrueckii* has been shown to accelerate wound closure and enhance collagen organization by modulating IL-17/NF-κB and TGF-β/Smad signaling pathways [174]. This probiotic upregulates GCLC and GSS, two enzymes essential for GSH biosynthesis, thereby reinforcing antioxidant capacity, stimulating keratinocyte proliferation, and promoting ECM remodeling [174].

Collectively, these findings highlight that precise redox regulation is indispensable for efficient wound healing. Therapeutic interventions employing ROS-scavenging hydrogels, antioxidant biomaterials, or probiotic-derived metabolites have shown significant enhancement in re-epithelialization, underscoring redox modulation as a viable therapeutic axis in wound management.

### 5.5. Immunometabolic and Hypoxic Adaptations

Wound microenvironments are inherently hypoxic, a condition that stabilizes HIF-1α and induces metabolic reprogramming in immune and stromal cells [34]. Under low oxygen tension, macrophages undergo a shift from oxidative phosphorylation to aerobic glycolysis, a phenomenon reminiscent of the Warburg effect, promoting M1 polarization and pro-inflammatory gene expression [75]. This metabolic adaptation enhances bactericidal activity but can also perpetuate inflammation when dysregulated.

Conversely, commensal-driven IL-10 signaling favors M2 macrophage polarization, which is central to tissue repair. IL-10 activates the JAK2/STAT3 pathway, promoting collagen synthesis and angiogenesis [175]. M2 macrophages secrete transforming growth factor-β (TGF-β) and connective tissue growth factor (CTGF), which, via AKT, ERK1/2, and STAT3 signaling, stimulate fibroblast proliferation, collagen deposition, and vascular remodeling—core features of the proliferative phase of wound healing [176].

In contrast, dysbiotic microbial consortia can accentuate glycolytic flux and promote succinate accumulation, reinforcing M1 macrophage polarization and nitric oxide production, thereby perpetuating chronic inflammation [177,178]. These metabolic shifts are governed by the interplay between AMPK, mTOR, and HIF-1α, representing an integrative regulatory hub that determines whether wounds progress toward resolution and regeneration or stagnate in inflammatory persistence [179].

### 5.6. Integrative View

The molecular interplay between the microbiota and host epithelia establishes a dynamic signaling continuum that bridges innate immune recognition, redox regulation, and metabolic adaptation [180]. In a balanced state, these interactions create a self-regulating ecosystem that coordinates inflammation, microbial containment, and tissue repair [181]. However, perturbations—such as antibiotic overuse, persistent hypoxia, or oxidative stress—disrupt this equilibrium, shifting the system toward chronic inflammation and impaired healing [182].

By integrating microbial, immunologic, and metabolic dimensions, a holistic understanding of this microbe–host interface provides a conceptual foundation for microbiome-targeted therapeutics. Strategies that modulate AMP expression, quorum-sensing activity, Nrf2-mediated redox control, or immunometabolic signaling hold promise for restoring tissue homeostasis and promoting effective wound closure [183,184,185]. These insights collectively redefine the microbiota not as passive inhabitants, but as active molecular architects of wound repair.

## 6. Wound Healing and Skin Microbiota

Following the preceding discussion on chronic wound pathophysiology—particularly the interplay among infection, inflammation, and biofilm persistence—emerging evidence highlights the skin microbiota as a critical determinant of wound outcomes. This intricate microbial ecosystem maintains immune homeostasis, modulates inflammatory signaling, and coordinates tissue regeneration. Disruption of this balance, or dysbiosis, skews immune responses, perpetuates inflammation, and impedes re-epithelialization—hallmarks of chronic non-healing wounds.

As the body’s largest organ, the skin functions not only as a physical barrier but as a highly dynamic immunological interface, integrating microbial and host-derived cues. It harbors a diverse consortium of microorganisms—collectively termed the skin microbiota—including bacteria, fungi, viruses, and arthropods [118]. Most members exist in mutualistic or commensal relationships, contributing to epithelial integrity, immune regulation, and microbial competition [48,186].

Maintaining a slightly acidic pH (4–6) is essential for preserving microbiota composition and activating epidermal lipid-processing enzymes, which stabilize the barrier [187,188]. Within this microenvironment, commensals confer colonization resistance by producing antimicrobial metabolites that suppress opportunistic pathogens.

Among these, *Staphylococcus epidermidis* is the most well-studied commensal, exerting multiple pro-barrier and anti-pathogenic effects. It stimulates sphingomyelinase-mediated ceramide synthesis, strengthening the lipid matrix [189], and secretes antimicrobial peptides and the serine protease inhibitor Esp, which inhibit *S. aureus* colonization [190]. Through TLR2 activation on keratinocytes, *S. epidermidis* enhances host AMP production and regulates inflammation following injury via LTA–mediated modulation of cytokine signaling [23,191]. These molecular dialogues contribute to a balanced immune tone, preventing excessive inflammation while promoting tissue repair [192]. Consequently, the skin microbiome is now recognized as an integral component of wound biology and a promising target for microbiota-based therapeutic innovation [42].

Beyond bacteria, fungal, viral, and arthropod residents also influence skin homeostasis. Malassezia species dominate the skin mycobiome, acting as both commensals and opportunists in inflammatory dermatoses [42,193,194]. Demodex mites, common arthropod inhabitants, may transition from benign symbionts to pathogenic under immune imbalance [42,194]. The skin virome, though the least characterized, contributes to cutaneous immune regulation and microbial population dynamics [42]. Its role in wound healing remains a frontier of microbiome research.

### 6.1. The Abundant Bacteria Implicated in Wounds

Metagenomic sequencing, particularly 16S rRNA gene profiling, has revealed the extraordinary diversity of the skin microbiome, encompassing at least 19 phyla and over 1000 species [48]. The four dominant bacterial phyla—Actinobacteria, Firmicutes, Proteobacteria, and Bacteroidetes—comprise the majority of cutaneous residents [48]. Actinobacteria typically dominate (≈52%), followed by Firmicutes and Proteobacteria, though their relative abundance varies across skin topographies [48,195]. At the genus level, *Staphylococcus*, *Cutibacterium* (formerly *Propionibacterium*), and *Corynebacterium* form the core microbiota [48].

During wounding, barrier disruption exposes nutrient-rich tissue, creating a niche for pathogen colonization. Chronic wounds are particularly vulnerable to biofilm-forming opportunists, whose persistence drives inflammation and tissue breakdown [196]. The most clinically significant include *Staphylococcus aureus* and *Pseudomonas aeruginosa*, often co-isolated and capable of synergistic interactions that enhance biofilm density and immune evasion [127,197,198]. However, antagonistic relationships also occur—*P. aeruginosa* can suppress *S. aureus* through inhibitory metabolites [199,200]—illustrating the complexity of polymicrobial dynamics.

Anaerobes such as Bacteroides fragilis are frequently found in deep or ischemic diabetic ulcers, emphasizing the oxygen gradient shaping chronic wound microbiota [201]. Other pathogens—*Streptococcus pyogenes*, *Klebsiella pneumoniae*, *Proteus* spp., *Enterococcus* spp., and *E. coli*—contribute to biofilm resilience, immune evasion, and proteolytic tissue degradation [126]. These organisms collectively determine wound chronicity, influencing both local immunity and systemic outcomes. Representative commensal and pathogenic bacteria, together with their experimentally defined effects on wound healing, are summarized in Table 1.

### 6.2. Coagulase-Negative Staphylococci (CoNS)

CoNS—including *Staphylococcus epidermidis*, *S. hominis*, and *S. haemolyticus*—represent dominant and functionally diverse constituents of the human skin flora. They play an essential role in maintaining epithelial–immune equilibrium but can transition into opportunistic pathogens when the cutaneous barrier is compromised or following invasive medical procedures [219,220]. This dualistic nature makes CoNS both protectors of skin homeostasis and contributors to disease under dysregulated conditions.

Among them, *S. epidermidis* is the most extensively studied species. It promotes IL-17A–dependent immune surveillance [202] and generates trace amines that act on adrenergic receptors to stimulate epithelial proliferation and repair [221]. However, this bacterium’s behavior is highly context dependent. Clinical isolates derived from chronic ulcers exhibit enhanced biofilm formation and induce proinflammatory cytokines—including IL-1β, IL-6, and IL-8—resulting in persistent inflammation and impaired wound closure [222]. These opposing phenotypes highlight the contextual plasticity of CoNS, reflecting their capacity to oscillate between commensalism and pathogenicity depending on host immunity and environmental conditions.

*Staphylococcus hominis*, another abundant skin commensal, further exemplifies this adaptive spectrum. It secretes autoinducing peptides (AIPs) that inhibit *S. aureus* quorum sensing and virulence [207] and produces antibiotics with potent antimicrobial activity—some of which have progressed to Phase I clinical evaluation as topical probiotics for atopic dermatitis [223]. Beyond antimicrobial defense, *S. hominis* abundance has been correlated with markers of skin health such as enhanced hydration, smaller pore size, and reduced wrinkle formation, suggesting its participation in maintaining structural and biochemical barrier integrity [206]. Yet, like other CoNS, *S. hominis* can act opportunistically in immunocompromised hosts. Biofilm formation on medical devices or compromised skin may lead to bacteremia, endocarditis, or meningitis, representing a major clinical concern [224,225,226]. Within wounds, these biofilms hinder healing by sustaining inflammation and blocking progression from the inflammatory to proliferative phase [209,227,228,229,230,231].

CoNS species generally display low intrinsic virulence; for instance, *S. saprophyticus* and *S. lugdunensis* rarely cause disease in immunocompetent individuals [219]. However, their biofilm-forming capability and antimicrobial tolerance complicate treatment once infection is established. Glycopeptides such as teicoplanin remain the first-line therapy, especially against methicillin-resistant strains, while linezolid and daptomycin demonstrate strong efficacy owing to low minimum inhibitory concentrations [232]. Combination antibiotic regimens are often preferred for persistent or device-associated infections. A clinical study on CoNS isolated from neonates revealed that combining vancomycin with oxacillin and gentamicin reduced biofilm density by 58.3% compared with monotherapies, highlighting the benefit of synergistic targeting [233]. Nonetheless, further research into the phylogeny, ecology, and adaptive mechanisms of CoNS is essential to inform both prevention and therapeutic design [219]. 

#### 6.2.1. *Staphylococcus epidermidis*

*Staphylococcus epidermidis* is a commensal CoNS and a keystone species of the cutaneous microbiota, integral to immune priming, barrier maintenance, and microbial competition [234,235]. It exhibits probiotic-like properties, largely through the modulation of host signaling pathways. For example, *S. epidermidis* secretes sphingomyelinase, which enhances ceramide synthesis, fortifying the epidermal lipid barrier and preventing transepidermal water loss [189]. It also activates mucosal-associated invariant T (MAIT) cells and CD8⁺ T cells, accelerating re-epithelialization and repair [203,236]. 

Therapeutically, *S. epidermidis*–derived molecules show translational potential. Topical bacterial extracts have been shown to improve incisional wound healing in rat models [204], and ex vivo human studies—using both diabetic and non-diabetic skin—demonstrated that *S. epidermidis* lysates stimulate keratinocyte migration and re-epithelialization, suggesting a role in the treatment of chronic wounds such as diabetic foot ulcers [205].

Despite these benefits, the pathogenic potential of *S. epidermidis* emerges under dysbiotic conditions. Strain-specific virulence, particularly biofilm formation, is linked to elevated proinflammatory cytokine production (IL-1β, IL-6, IL-8) and delayed tissue regeneration [222]. Murine studies have shown that cytokine inhibition in biofilm-associated infections restores epithelial repair, underscoring the therapeutic relevance of targeting host–pathogen immune crosstalk [237]. Therefore, *S. epidermidis* embodies a dual paradigm—protective and reparative under homeostatic conditions, yet pathogenic and inflammatory in chronic or biofilm-dominated environments. Dissecting this strain-dependent heterogeneity is essential to harness its full potential in microbiome-based wound therapy.

#### 6.2.2. *Staphylococcus hominis*

*Staphylococcus hominis* is the second most prevalent CoNS species on healthy human skin, particularly concentrated in apocrine-rich regions such as the axillae [224]. While traditionally recognized for contributing to body odor through conversion of odorless precursors into volatile sulfur compounds (e.g., 3-methyl-3-sulfanylhexan-1-ol, 3M3SH), it also plays a beneficial immunological role in maintaining microbial equilibrium [224].

#### 6.2.3. *Staphylococcus aureus*

*Staphylococcus aureus* is a Gram-positive, coagulase-positive pathogen that colonizes the skin and nasal passages but readily transitions into an invasive agent following barrier disruption. Since the emergence of methicillin-resistant strains (MRSA) in the 1960s, it has become one of the most significant causes of hospital- and community-acquired infections [238].

Pathogenicity is multifactorial, involving superantigens, cytotoxins, and biofilm formation. At low concentrations, superantigens can paradoxically suppress IL-17 signaling and neutrophil recruitment, thereby attenuating local inflammation [1]. Conversely, high systemic levels provoke immune hyperactivation and tissue necrosis [1]. In chronic wounds, *S. aureus* induces sustained expression of IL-1β, IL-6, TNF-α, and CXCL-1, leading to prolonged inflammation and delayed re-epithelialization [1].

Biofilm formation further enhances persistence by limiting antibiotic penetration and impairing keratinocyte migration [209,210]. In conditions such as atopic dermatitis, disease flares coincide with *S. aureus* overgrowth and depletion of protective CoNS, highlighting the importance of microbial balance in skin health [239,240,241]. These findings collectively position *S. aureus* as both a driver and consequence of dysbiosis, underscoring the need for anti-biofilm and immune-modulating strategies to improve clinical outcomes in chronic wound care.

#### 6.2.4. *Pseudomonas aeruginosa*

*P. aeruginosa* is a Gram-negative opportunistic pathogen frequently implicated in chronic and nosocomial wound infections, particularly in immunocompromised or diabetic patients [242]. Its large genome and regulatory flexibility underpin its metabolic adaptability and capacity for persistence in hostile environments [242,243].

Pathogenesis involves potent inflammatory activation and biofilm formation. Acute infections are characterized by upregulation of IL-1α, IL-1β, TNF-α, and IL-8, which recruit neutrophils and promote collateral tissue damage [212,244]. Biofilm development—one of its defining traits—creates a protective matrix that limits antibiotic diffusion and immune clearance [97,245,246]. Compared with *S. aureus*, *P. aeruginosa* often penetrates deeper wound layers, compounding therapeutic difficulty [242].

Interestingly, certain *P. aeruginosa* strains, such as PAO1, can transiently promote early wound closure by inducing TNF-α–mediated angiogenesis and epithelial proliferation, suggesting a context-dependent immunomodulatory effect [247,248]. Nevertheless, its inherent antibiotic resistance and biofilm resilience remain formidable challenges. Deciphering the molecular dialogue between host and pathogen, particularly through integrative omics approaches, will be crucial for developing targeted interventions that restore wound homeostasis [242].

#### 6.2.5. *Streptococcus pyogenes*

*Streptococcus pyogenes* (Group A *Streptococcus*, GAS) is a β-hemolytic, Gram-positive bacterium responsible for a broad range of infections, from pharyngitis to necrotizing fasciitis [249]. In wound pathology, it contributes to both acute and chronic infections, particularly among immunocompromised or diabetic individuals [250,251]. Its virulence is mediated by M-protein, extracellular toxins, and a polysaccharide capsule, which together prevent phagocytic clearance and sustain inflammation [250,251,252].

Therapeutically, natural bioactive compounds have shown promise in mitigating *S. pyogenes*–associated damage. Honey–chitosan hydrogels display strong antimicrobial and pro-healing activity, significantly reducing bacterial load and accelerating wound repair in murine burn models [253,254,255]. Moreover, nanoparticle-based dressings are emerging as next-generation alternatives, providing controlled antimicrobial release and enhanced biocompatibility [256]. Such integrated strategies emphasize the potential of biomaterial-assisted wound therapy that simultaneously targets infection and promotes tissue regeneration.

#### 6.2.6. *Lactobacilli*

Members of the genus *Lactobacillus* are Gram-positive, lactic acid–producing bacteria within the phylum Firmicutes, known for their probiotic, immunomodulatory, and barrier-strengthening functions [257,258,259]. They naturally inhabit mucosal sites including the oral, intestinal, and urogenital tracts [260]. Topical application of lactobacilli has shown significant benefit in wound repair. *Lactobacillus plantarum*, for example, disrupts *P. aeruginosa* biofilms and enhances re-epithelialization by promoting AMP production and modulating cytokine expression [261,262]. Both live and heat-killed preparations have demonstrated efficacy, the latter emphasizing that postbiotic components alone can stimulate fibroblast proliferation and immune regulation [218].

Comparative studies reveal strain-specific differences: *L. casei*, *L. acidophilus*, and *L. rhamnosus* exhibit strong antimicrobial and reparative effects, whereas *L. delbrueckii* displays limited activity [263,264]. Consequently, precision in strain selection and mechanistic profiling remains critical for clinical translation [261].

#### 6.2.7. *Lactobacillus plantarum*

*Lactobacillus plantarum* is a facultatively anaerobic or microaerophilic species with broad antimicrobial, antioxidant, and immunomodulatory activity [265,266,267]. It adheres to epithelial surfaces via mannose-specific adhesins, competitively excluding pathogens and secreting antimicrobial metabolites such as lactic acid, benzoic acid, hydrogen peroxide, and bacteriocins [268,269]. Its inhibitory spectrum includes *P. aeruginosa*, *Listeria monocytogenes*, *E. coli*, and *Enterococcus faecalis* [270,271], with certain strains showing efficacy against multidrug-resistant isolates [272].

Beyond its antimicrobial profile, *L. plantarum* reduces oxidative stress by lowering malondialdehyde (MDA) and reactive oxygen species (ROS) levels while suppressing TNF-α, IL-6, and IL-1β production [217,218,273]. In diabetic wound models, it mitigates advanced glycation end-product (AGE)–induced NLRP3 inflammasome activation and pyroptosis, thereby restoring cellular homeostasis and accelerating closure [216,274]. These multifaceted effects position *L. plantarum* as a multifunctional probiotic capable of simultaneously modulating microbial, oxidative, and immune axes of wound repair. Further studies should delineate strain-specific activity, optimize delivery systems, and validate its clinical efficacy through controlled trials.

#### 6.2.8. *Escherichia coli*

*Escherichia coli* is a Gram-negative, facultative anaerobe and a predominant commensal of the human gut microbiota [275,276]. While non-pathogenic strains coexist harmlessly, pathogenic variants exploit inflammatory environments to delay healing through biofilm formation, immune evasion, and tissue damage [277,278]. Chronic wound isolates frequently exhibit biofilm formation within an extracellular polymeric matrix that shields them from immune clearance and antimicrobial penetration, thereby sustaining infection [279,280]. Elevated bacterial loads (>10^5^ CFU/g tissue) correlate strongly with delayed closure and increased antibiotic resistance [99,277,279].

Conversely, probiotic strains such as *E. coli* Nissle 1917 lack virulence factors and possess tissue-reparative properties [281]. This strain enhances mucosal barrier integrity, activates AKT–ERK1/2 signaling, and promotes EGFR-mediated epithelial migration [281]. Engineered derivatives expressing EGF further amplify wound closure, highlighting the therapeutic promise of genetically optimized probiotic *E. coli* in reparative medicine [281].

### 6.3. Other Bacteria Implicated in Wounds

Apart from the aforementioned bacteria, there is another bacterium, *Enterococcus faecalis*, that is implicated in wounds, although it is relatively less abundant.

#### *Enterococcus* *faecalis*

*Enterococcus faecalis* is a Gram-positive, facultative anaerobe residing in the gastrointestinal tract, oral cavity, and upper respiratory mucosa [282]. Though typically commensal, it has emerged as a clinically significant pathogen in bacteremia, endocarditis, and wound infections [282]. Within chronic wounds, *E. faecalis* disrupts tissue repair by upregulating TGF-β1 and downregulating PDGF-A, impairing fibroblast function and extracellular matrix deposition [213].

At high bacterial burdens, *E. faecalis* alters macrophage polarization toward an anti-inflammatory phenotype, leading to persistent infection and delayed closure [213]. The organism’s ability to invade fibroblasts and express multiple peptide resistance factors further facilitates immune evasion and chronic persistence [214]. Although less abundant than *S. aureus* or *P. aeruginosa*, its pathophysiological impact is substantial. Therefore, *E. faecalis* should be recognized as a clinically relevant and mechanistically complex contributor to chronic wound microbiota, where it modulates immune, cellular, and structural components of repair.

### 6.4. Relationship Between Skin Microbiome and Wound Healing

The skin microbiome plays a central regulatory role in cutaneous wound repair, influencing outcomes through its composition, diversity, and functional activity [283]. Following injury, the breach of the epithelial barrier exposes nutrient-rich tissues that can support either tissue repair processes or pathogenic overgrowth, depending on the balance between commensal and opportunistic microorganisms [284].

Commensal bacteria actively promote wound resolution through several mechanisms. They modulate early innate immune responses, induce keratinocyte-derived cytokines, and activate T-cell–independent repair pathways that accelerate epithelial recovery [285]. In parallel, commensals prevent opportunistic colonization through competitive exclusion and the secretion of AMPs and enzymes [286]. For instance, *S. epidermidis* produces AMPs and the serine protease inhibitor Esp, both of which inhibit *S. aureus* colonization, while *S. capitis* interferes with *S. aureus* virulence gene expression, thereby suppressing pathogenic dominance [118,190].

The composition and diversity of the wound microbiota are closely correlated with healing trajectory [287]. A diverse and balanced microbial community promotes epithelial migration, angiogenesis, and immune homeostasis, whereas dysbiosis, often characterized by *S. aureus* overrepresentation, drives chronic inflammation and impaired closure [287]. Pathogenic dominance contributes to tissue damage through the secretion of cytotoxins, hemolysins, and phenol-soluble modulins derived from organisms such as *S. aureus* and *S. epidermidis*, which disrupt endothelial integrity, induce oxidative stress, and facilitate immune evasion [288,289]. 

Traditional antimicrobial interventions—particularly broad-spectrum topical or systemic antibiotics—can further disturb microbial equilibrium, eliminating beneficial commensals and promoting antibiotic-resistant biofilm-forming strains [288]. Biofilms, in turn, impede antibiotic diffusion, suppress immune clearance, and maintain bacterial persistence through adaptive gene expression [288,290].

To counter these limitations, emerging microbiome-aware wound therapies aim to restore homeostasis while controlling infection. Promising strategies include topical probiotics and prebiotics, engineered antimicrobial peptides, and selective wound dressings that preserve beneficial microbial species while targeting pathogens [262]. Preserving or reestablishing a balanced microbiota is increasingly recognized as equally critical to wound resolution as pathogen eradication, representing a paradigm shift toward ecological and host-supportive wound management.

### 6.5. Interaction Between Perforin-2 and Skin Microbiota in Wound Healing

A recent focus in cutaneous immunology concerns the interaction between Perforin-2 (P-2)—a pore-forming innate immune effector—and the skin microbiota during tissue repair [291]. Keratinocytes, which form the primary epithelial barrier, constitutively express P-2 as part of their antimicrobial arsenal [284]. Upon bacterial challenge, P-2 localizes to endosomal membranes, where it fuses with phagosomes containing intracellular bacteria, forming pores that compromise microbial membranes and enable intracellular clearance [284].

The antimicrobial function of P-2 is both strain-specific and context-dependent, modulated by host–microbiome interactions. Commensals such as *Staphylococcus epidermidis* can enhance P-2 expression through activation of γδ T cells, thereby strengthening keratinocyte-mediated defense against intracellular pathogens like *S. aureus* [292]. In this manner, commensal microorganisms indirectly augment epidermal immunity and facilitate efficient wound healing.

Conversely, *S. aureus*—a leading agent in chronic wound infections—has evolved mechanisms to evade or suppress P-2–mediated immunity [284]. It can downregulate P-2 expression in both hematopoietic and non-hematopoietic cells, impairing intracellular bacterial clearance and promoting infection persistence [284]. Ex vivo human wound models have demonstrated that *S. aureus*–mediated P-2 suppression correlates with delayed epithelial closure and chronic inflammation [284]. Furthermore, antibiotic-resistant strains exhibit enhanced capacity to subvert P-2 activity, exacerbating immune escape [293].

Although P-2 activation alone may not completely eliminate *S. aureus*, experimental evidence indicates that it restricts bacterial dissemination and reduces infection severity in murine models [294]. Thus, therapeutic strategies that enhance commensal-driven P-2 upregulation or pharmacologically modulate P-2 signaling could provide a novel means of restoring antimicrobial equilibrium in chronic wounds. Understanding the molecular mechanisms by which commensals potentiate P-2 activity—and how pathogens circumvent it—will be pivotal in developing next-generation microbiome-based immunotherapies, particularly in the context of rising antimicrobial resistance.

## 7. Therapeutic and Diagnostic Applications of the Wound Microbiome

Growing insight into host–microbiota crosstalk has redefined wound management, shifting the paradigm from broad antimicrobial eradication toward ecological re-balancing and molecular modulation of repair processes. Next-generation interventions now target both the microbial ecosystem and the host immune–metabolic network to restore barrier integrity, resolve chronic inflammation, and promote functional regeneration.

### 7.1. Probiotics and Postbiotics

Probiotics—defined by the International Scientific Association for Probiotics and Prebiotics as “live microorganisms which, when administered in adequate amounts, confer a health benefit on the host” [295] —have emerged as promising bioactive agents in cutaneous wound management. Their therapeutic potential stems from their ability to modulate immune and epithelial responses, enhance angiogenesis, and competitively inhibit pathogen colonization, thereby creating a microenvironment conducive to repair [295,296]. Importantly, probiotic efficacy is strain-specific and context-dependent, reflecting variations in microbial metabolism, host interaction, and local immune tone [297]. Topical and hydrogel-embedded formulations containing *Lactobacillus plantarum*, *L. rhamnosus*, and *L. casei* accelerate re-epithelialization and neovascularization in both *in vivo* and *in vitro* models [298,299,300]. These effects are mediated through microbial metabolites that activate VEGF and TGF-β signaling—key drivers of endothelial proliferation, ECM deposition, and tissue remodeling [300]. In parallel, these strains suppress *Staphylococcus aureus* colonization and down-regulate pro-inflammatory cytokines (IL-6, TNF-α), thereby promoting immune resolution [301]. Clinical and pre-clinical data indicate that probiotic supplementation shortens healing time and reduces grafting requirements by accelerating closure [302]. Oral administration also enhances collagen synthesis and dermal tensile strength, underscoring systemic immunometabolic contributions to cutaneous regeneration [283].

At the systemic level, specific probiotic strains enhance natural-killer cell activity, increase antibody production, and rebalance cytokine profiles toward anti-inflammatory, tissue-protective phenotypes [303]. *Escherichia coli* Nissle 1917, a non-pathogenic commensal, activates AKT–ERK1/2 signaling to promote epithelial proliferation [213]; when engineered to express EGF, it further accelerates keratinocyte migration through EGFR activation [281].

Postbiotics—per the ISAPP consensus, “preparations of inanimate microorganisms and/or their components that confer a health benefit on the host”—offer a complementary strategy to live-strain therapy [304]. In wound care, the evidence base for postbiotics remains emerging relative to probiotics, with most data from *in vitro* and preclinical models [305]. Nevertheless, cell-free supernatants, heat-killed preparations, exopolysaccharides, and microbially derived metabolites can modulate keratinocyte and fibroblast programs and bolster antimicrobial defense [306]. Among these, short-chain fatty acids (e.g., butyrate, propionate) have been shown in skin-relevant systems to influence fibroblast proliferation and matrix-gene expression via GPR43-dependent AMPK signaling and histone deacetylase inhibition, linking microbial metabolism to epigenetic control of repair pathways (predominantly preclinical evidence) [307,308]. Furthermore, topical postbiotic preparations derived from *Lactobacillus fermentum*, *Lactobacillus reuteri*, and *Bacillus* subtilis var. natto, formulated in a cold-cream base, are being explored as novel wound therapeutics. In treated models, the *L. reuteri* postbiotic group demonstrated earlier complete re-epithelialization and showed no detectable cutaneous inflammation compared with controls [309]. Taken together, probiotics and postbiotics function as multi-target modulators of immune tone, epithelial regeneration, and pathogen control; however, larger, well-controlled clinical trials—particularly for postbiotics—are needed to define preparations, dosing, delivery systems, and indications across wound etiologies.

### 7.2. Quorum-Sensing Inhibitors and Anti-Biofilm Strategies

Beyond rebalancing the microbiome through probiotic and postbiotic therapies, a complementary strategy seeks to silence pathogenic communication directly. Chronic wound infection is sustained by biofilm formation and QS–driven virulence, which collectively enable pathogens to coordinate adhesion, immune evasion, and antibiotic tolerance [310].

Emerging evidence indicates that natural flavonoids such as quercetin and baicalin disrupt *P. aeruginosa las/rhl* circuits, thereby reducing elastase and rhamnolipid synthesis [311,312]. Similarly, synthetic peptides antagonizing the *Staphylococcus aureus agr* system block α-toxin production and inhibit biofilm maturation [313].

Enzymatic dispersal agents—including DNase I and dispersin B—further degrade extracellular matrices, enhancing antibiotic diffusion and immune accessibility [314]. Nanocarrier-based co-delivery systems that combine QS inhibitors with antibiotics achieve synergistic biofilm inhibition while minimizing cytotoxicity [315]. Together, these interventions exemplify a paradigm shift toward pathogen silencing rather than indiscriminate killing, thereby preserving commensal balance while attenuating virulence.

Practical Considerations. Biofilm heterogeneity, inducible tolerance, restricted drug diffusion within dense matrices, and the potential emergence of resistance to QS inhibitors or dispersal enzymes remain major translational hurdles [316]. To overcome these barriers, research is increasingly focused on device-adapted and wound-responsive delivery systems capable of penetrating complex microbial communities and releasing anti-QS agents in a controlled manner [316]. Collectively, quorum-sensing inhibition and biofilm dispersal represent precision strategies that suppress virulence while maintaining the ecological equilibrium essential for effective wound resolution.

### 7.3. Bacteriophage-Based Therapeutics

Bacteriophage therapy offers highly specific, self-amplifying antibacterial action that spares beneficial microbiota. Phages targeting *S. aureus* and *Acinetobacter baumannii* demonstrate promising efficacy in animal and compassionate human applications [317]. Combination therapy with antibiotics or probiotics enhances microbial stability and minimizes recurrence [318].

Although challenges remain—such as resistance evolution and manufacturing standardization—phage-derived endolysins show promise as broad-spectrum, anti-biofilm enzymes [319]. Modern phage cocktails and engineered lysins therefore represent precision-guided tools aligned with ecological principles of targeted pathogen control.

### 7.4. Engineered Antimicrobial Peptides and Peptidomimetics

AMPs bridge innate defense and regenerative signaling. Through rational design and computational optimization, new short cationic peptides have been engineered to selectively disrupt microbial membranes while enhancing keratinocyte migration and proliferation [320]. Hybrid delivery systems coupling AMPs to nanoparticles, hydrogels, or collagen scaffolds ensure sustained release and improved peptide stability.

Synthetic LL-37 derivatives can simultaneously suppress *P. aeruginosa* QS pathways and activate EGFR–STAT3 signaling, linking antimicrobial efficacy with epithelial restitution [321,322,323]. These bioresponsive constructs epitomize the transition from purely bactericidal approaches to dual-function, pro-healing biomolecules that target infection and stimulate repair in tandem.

### 7.5. Microbiome-Informed Diagnostics and Omics-Driven Stratification

Advances in multi-omics technologies—including 16S rRNA sequencing, metagenomics, metatranscriptomics, and metabolomics—have transformed wound-ecosystem profiling [324,325]. Integration of microbial and host transcriptomes reveals reciprocal regulation between community composition and immune-gene expression, enabling predictive modeling of healing trajectories [326,327].

Portable sequencing platforms such as Oxford Nanopore MinION now enable near-real-time bedside analysis, reducing empirical antibiotic use and guiding personalized therapy [328,329]. Concurrently, metabolomic biomarkers—short-chain fatty acids, indole derivatives, and siderophores—correlate with wound chronicity and serve as non-invasive indicators of dysbiosis [330,331].

Integrating these omics-level biomarkers with machine-learning algorithms enables individualized microbial risk stratification and real-time precision guidance for wound management.

### 7.6. Integration into Clinical Practice

Translating microbiome-targeted approaches from laboratory innovation to routine wound care necessitates rigorous standardization of sampling, sequencing, and analytical methodologies. Recent findings from the “Wound-BIOME” project demonstrated that standardized wound swab sampling can indeed yield high-quality material for OMICS analyses, supporting this translational effort [332]. Regulatory frameworks must evolve to encompass microbe-based medicines such as live biotherapeutic products (LBPs), which, unlike conventional probiotics, are designed to treat or prevent disease but pose unique regulatory challenges related to quality, safety, stability, and patient-specific microbiome variability [333].

Future directions point toward convergent regenerative medicine, integrating microbiome modulation, stem-cell-derived exosomes, and bioactive scaffolds to restore both tissue architecture and microbial equilibrium [334,335,336].

This integrative paradigm reframes chronic wounds as eco-immunological disorders, wherein microbial imbalance and immune dysregulation are co-drivers of pathology [2]. The ultimate objective is a precision-medicine model that harmonizes host and microbiota for durable, functionally complete healing.

## 8. Biofilms

Biofilms are highly organized microbial communities encased in a self-produced EPS matrix, which provides mechanical stability and protects cells from environmental stresses, including desiccation, host immune defenses, and antimicrobial agents [337]. Beyond physical protection, the EPS matrix promotes horizontal gene transfer, metabolic cooperation, and collective antibiotic tolerance, enabling microbial communities to persist under hostile conditions [325].

Within biofilms, distinct aerobic and anaerobic microenvironments form, driven by gradients in nutrients, oxygen, and redox potential [337]. These micro-niches enable selective enrichment of bacterial species and spatial stratification of metabolic activity, reflecting both nutrient diffusion and interspecies signaling [337]. 

At infection sites, microbes exist predominantly in two phenotypic states: planktonic (free-living) or biofilm-associated (attached or aggregated). Planktonic cells can transition into biofilms via a regulated process involving surface sensing, genetic reprogramming, and EPS production [338]. The EPS matrix—comprising water-soluble polysaccharides, proteins, extracellular DNA (eDNA), and water-insoluble compounds—undergoes dynamic compositional changes during biofilm development and in response to environmental or interspecies cues [339].

Biofilm formation and maintenance rely on a complex interplay of genetic and environmental signals. The EPS scaffold fosters microbial persistence, metabolic cooperation, and communication through quorum sensing. Coexisting species within polymicrobial biofilms may alter EPS composition to enhance collective survival [339].

Clinically, biofilms are a major contributor to chronic wound pathology, present in over 60% of chronic wound infections compared to ~6% of acute wounds [340]. They impair healing by compromising skin barrier function, disrupting evaporative water regulation, and evading host innate immunity [338]. Enzymatic degradation of the extracellular matrix further weakens the wound bed, and even epithelialized wounds can remain biofilm-colonized, exhibiting compromised matrix integrity and a predisposition to recurrence [338].

Given their central role in chronic wound persistence, targeted anti-biofilm strategies are urgently needed. Effective interventions should extend beyond antibacterial agents to also target fungal and protozoan biofilm components, aiming to disrupt the EPS matrix while restoring host tissue function without impeding wound closure. Future approaches should integrate microbial eradication with immunomodulatory and regenerative therapies to optimize wound healing outcomes. 

## 9. Analytical Advances, Methodological Challenges, and Future Perspectives in Wound–Microbiome Research

Despite remarkable advances in profiling the skin and wound microbiota, the field has yet to transition fully from descriptive taxonomic cataloging to functional and translational precision. Several methodological, biological, and clinical constraints continue to impede the realization of microbiome-guided wound management.

### 9.1. Overview of Microbiome Identification Techniques

Accurate identification of the skin microbiota is essential for elucidating its role in health, disease pathogenesis, and wound repair [92,341]. Early studies employed electrophoretic fingerprinting methods such as denaturing and temperature gradient gel electrophoresis (DGGE, TGGE) to generate community-level profiles, though these lacked taxonomic and functional resolution. The advent of culturomics, metagenomics, next-generation sequencing (NGS), and whole-genome sequencing (WGS) has transformed the field, providing high-resolution insights into microbial ecology, function, and dynamics [342].

Culture-based methods remain indispensable for isolating viable organisms and determining antimicrobial susceptibility; however, they underestimate microbial diversity since many fastidious and anaerobic taxa remain uncultivable under standard laboratory conditions [343]. In contrast, molecular-based techniques such as metagenomics, NGS, and WGS enable simultaneous detection of bacteria, fungi, viruses, and archaea, achieving superior taxonomic resolution and functional profiling [344,345,346]. These approaches have revealed the complex polymicrobial nature of chronic wounds, identifying microbial drivers of delayed healing, immune modulation, and antimicrobial resistance [261,347]. Ongoing advances in long-read sequencing accuracy, bioinformatics, and multi-omics integration are bridging the gap between discovery and clinical application, establishing the foundation for precision microbiome diagnostics and therapeutics.

### 9.2. Culture-Dependent Methods and Culturomics

Traditional culture-based microbiology remains indispensable for clinical microbiome studies, enabling the isolation of viable organisms and assessment of antimicrobial susceptibility [221,348]. However, many skin-associated microorganisms—particularly anaerobes and nutritionally fastidious species—require specialized environmental and nutritional conditions not replicated in standard laboratory settings [349].

To address these limitations, culturomics has emerged as a high-throughput cultivation approach employing diverse growth conditions—ranging from aerobic to strictly anaerobic—combined with advanced identification techniques such as MALDI-TOF mass spectrometry and 16S rRNA gene sequencing [350]. This strategy has significantly expanded our understanding of microbial diversity; for instance, culturomics of African skin microbiota revealed 71 previously uncharacterized bacterial taxa, including seven novel species, thereby increasing known diversity by 14% [351]. Despite these advances, culturomics remains labor-intensive and time-consuming, emphasizing the value of integrating it with genomic and metagenomic analyses to achieve a comprehensive view of the wound microbiome.

### 9.3. Metagenomic Sequencing

Metagenomic sequencing provides superior taxonomic resolution and functional insights into microbial communities compared to traditional culture-based methods [221]. It is particularly valuable for characterizing bacteria within biofilms, which are common in chronic wounds and often evade detection by conventional techniques [352]. As a result, the wound microbiome is now recognized as more diverse and complex than previously understood [221].

Shotgun metagenomics—one of the most widely used approaches—sequences the entire DNA content of a sample, enabling an unbiased and comprehensive characterization of bacteria, fungi, viruses, and archaea. This method enhances detection of microbial diversity, improves species-level resolution, and allows functional profiling of microbial genes and metabolic pathways [353,354]. Spatial variability in skin microbiota has been demonstrated through this technique; for example, distinct bacterial communities have been observed across different facial regions, with *Cutibacterium* species more abundant in the nasal area than on the forehead or cheeks [355]. Furthermore, differences in *Cutibacterium* phage abundance between healthy individuals and acne patients suggest a potential role for bacteriophages in modulating the skin microbiome [355,356].

Challenges remain, particularly due to the skin’s low microbial biomass and contamination from host DNA, which can hinder comprehensive functional analyses [357]. Recent advances in long-read sequencing technologies, such as PacBio SMRT and Oxford Nanopore, combined with improved bioinformatics pipelines, have mitigated these issues, allowing deeper functional characterization of microbial communities [357].

Clinically, metagenomic sequencing has shown promise in managing chronic, non-surgical wounds. It can be incorporated into diagnostics to document wound colonization and track microbial shifts during treatment [358,359]. Unlike targeted sequencing methods, metagenomics does not require prior amplification of specific genetic markers, enabling unbiased, species-level identification and comprehensive analysis of polymicrobial infections [360]. Longitudinal metagenomic studies are especially valuable for understanding how microbial populations evolve over time and influence wound healing trajectories [361].

### 9.4. Next-Generation Sequencing and Its Applications

NGS technologies have been instrumental in advancing the characterization of wound microbiota, providing detailed insights into the relationships between microbial composition, wound healing outcomes, and infection-related complications [362]. Beyond bacterial profiling, NGS can target internal transcribed spacer (ITS) regions to assess fungal communities, thereby expanding microbiome analysis to encompass non-bacterial organisms implicated in chronic wound pathophysiology [221].

A major strength of NGS is its capacity to detect antimicrobial resistance (AMR) genes, supplying clinicians with actionable data for infection management [363]. Depending on research objectives and sample characteristics, a range of NGS-based strategies can be employed: 16S rRNA gene sequencing for bacterial profiling, ITS sequencing for fungi, shotgun metagenomics for comprehensive community profiling, and sequencing-by-ligation platforms such as SOLiD for transcriptomic and gene expression analyses [364,365]. Microbiome profiles generated through NGS can even be leveraged to predict disease susceptibility, analogous to approaches in human genomics [344].

Despite these advantages, NGS is not without limitations. It requires significant computational resources and advanced bioinformatics expertise, while the absence of standardized protocols for sample processing, sequencing, and data interpretation hinders its routine integration into clinical diagnostics [344]. Current research efforts are directed toward improving methodological consistency and analytical pipelines to support the broader adoption of NGS in dermatology, wound microbiology, and microbiome-based precision medicine.

### 9.5. Whole Genome Sequencing

WGS is a comprehensive genomic approach that enables precise microbial strain identification and functional characterization of microbial communities [366]. In wound microbiology, WGS facilitates the assessment of wound bioburden and comparison of microbial profiles between healthy and wounded skin, revealing critical microbial factors that influence healing [221]. Comparative studies show that, relative to healthy skin, chronic wounds harbor increased abundances of anaerobes, Gram-positive cocci, and Gram-negative rods, with a concomitant reduction in typical skin commensals [367].

WGS delivers strain-level resolution, elucidating virulence mechanisms, therapeutic responses, and key genes involved in pathogenesis and survival [368,369]. Unlike metagenomic approaches that provide community-level overviews, WGS generates complete genomic profiles, enabling the distinction between pathogenic and commensal strains within the same species [370,371]. It also allows for the detection of antimicrobial resistance (AMR) genes and resistance-associated mutations, supporting precise monitoring of resistance patterns [372].

Despite its advantages, WGS faces practical challenges, including the need for high-quality microbial DNA from often low-biomass skin samples, substantial computational resources for data analysis, and relatively high costs compared to targeted sequencing [373]. Nonetheless, WGS has transformed our understanding of microbial genetic variation—detecting alterations from single nucleotide polymorphisms (SNPs) to large structural variants such as insertions, deletions, and chromosomal rearrangements [374].

When integrated with other omics approaches—such as proteomics, metabolomics, and transcriptomics—WGS provides a holistic view of microbial activity and its role in wound pathophysiology. For example, a multi-omics study of diabetic foot ulcers (DFUs) incorporating WGS identified pathogenic strains, AMR genes, upregulated inflammatory pathways, biofilm-associated proteins, and dysregulated metabolites implicated in impaired healing [375]. Such integrative analyses highlight the potential of WGS as a cornerstone technology for precision wound diagnostics and targeted therapeutics.

### 9.6. Whole 16S rRNA Gene Sequencing

Whole 16S rRNA gene sequencing (~1500 bp) is a powerful molecular tool for differentiating bacterial species and strains, offering substantially higher taxonomic resolution than partial sequencing of hypervariable regions [376,377]. By capturing the full gene, this approach enables detection of single-nucleotide polymorphisms and fine-scale differentiation between closely related taxa, while minimizing amplification bias associated with shorter fragments [378].

Traditional skin microbiome studies have often targeted specific hypervariable regions—such as V1–V3 or V3–V4—due to earlier sequencing limitations [244] While cost-effective, this strategy can compromise taxonomic accuracy and misrepresent microbial diversity [376]. Full-length sequencing mitigates these drawbacks and is particularly valuable for achieving species- and strain-level resolution. However, it requires advanced long-read platforms such as PacBio SMRT and Oxford Nanopore, which, despite enabling comprehensive analysis, may introduce platform-specific errors [376]. When constrained by limited DNA input or short-read platforms, V1–V3 remains a practical alternative, balancing resolution and efficiency, though it still falls short of full-length capabilities [379].

Challenges of whole 16S rRNA sequencing include bioinformatic complexity, incomplete reference databases, and the lack of standardized protocols, all of which can hinder reproducibility and cross-study comparability [376,380,381]. To address these issues, platforms such as QIIME2 and Mothur provide robust pipelines for sequence processing, error correction, and taxonomic classification [382]. Clinically, full-length 16S sequencing enhances infectious disease diagnostics by detecting unculturable or fastidious organisms, thereby guiding more precise antimicrobial therapy [383,384].

Both whole 16S rRNA sequencing and metagenomic approaches remain among the most widely used strategies for skin microbiome characterization. While they offer exceptional resolution and functional insights, they are limited by high costs, computational demands, and data analysis complexity [385]. Culture-dependent methods continue to provide value for isolating viable bacteria and assessing antimicrobial susceptibility, but their inability to detect non-culturable species restricts their scope [376,378]. Ongoing improvements in long-read sequencing technologies and bioinformatics are expected to further enhance accuracy, reproducibility, and clinical applicability—advancing our understanding of skin microbiome’s role in wound healing, infection, and disease progression.

### 9.7. Technical and Methodological Constraints

Despite these methodological advances, several technical challenges persist. Most studies still rely on amplicon-based 16S rRNA sequencing, which provides taxonomic information but limited functional insight and cannot distinguish live from dead or biofilm-resident cells. Sampling heterogeneity—including variations in swabbing depth, collection media, and sequencing platforms—further complicates cross-study comparability. Moreover, the lack of curated, wound-specific microbial databases hinders accurate annotation of novel taxa.

Shotgun metagenomics and metatranscriptomics offer deeper functional insights but require standardized sampling, high coverage, and advanced bioinformatics pipelines. Integration of multi-omics data—linking microbial genomes with host transcriptomes, proteomes, and metabolomes—holds promise for unraveling causal mechanisms. However, reproducible computational workflows and open-access data frameworks are urgently needed to ensure inter-study consistency.

### 9.8. Biological Complexity and Host Variability

The wound microbiome is shaped by diverse host factors, including age, comorbidities (e.g., diabetes, vascular disease), immune status, and genetic polymorphisms. Microbe–host interactions are context dependent: the same microbial taxa may promote healing in one individual yet delay repair in another, depending on oxygenation, inflammation, and immune tone. This concept of “pathogenicity by context” challenges traditional infection-versus-colonization paradigms.

Furthermore, the coexistence of bacteria, fungi, and viruses introduces multilayered interactions—cross-kingdom signaling, bacteriophage predation, and metabolic exchange—that remain incompletely characterized. Spatially resolved technologies such as imaging mass spectrometry, laser-capture microdissection, and spatial transcriptomics now enable simultaneous visualization of microbial and host molecules, advancing our understanding of microenvironmental heterogeneity in wound tissues.

### 9.9. Translational and Clinical Barriers

Despite encouraging preclinical findings, the translation of microbiome-based interventions—such as probiotics, phage therapy, and quorum-sensing inhibitors—remains limited [89]. Variability in wound type, microbial burden, and patient adherence complicates clinical trial design. Regulatory ambiguity surrounding live biotherapeutic products and genetically engineered phages further delays progress. Moreover, current infection-control and antibiotic stewardship programs rarely account for microbiome preservation, potentially exacerbating dysbiosis.

To bridge these gaps, clinical implementation of microbiome diagnostics requires standardized manufacturing, validated safety protocols, and harmonized regulatory frameworks. Integrating microbial profiling into wound-care guidelines will enable data-driven therapeutic decisions that balance eradication with ecological restoration.

### 9.10. Future Directions

Future research should prioritize:

1. Longitudinal multi-omics studies correlating microbial dynamics with host immune and metabolic signatures.
2. Functional modeling using 3-D organotypic skin systems and humanized-microbiota animal models to define causality.
3. Systems-biology frameworks integrating microbial networks, metabolite fluxes, and host signaling pathways.
4. Precision therapeutics, including synthetic microbial consortia, bioengineered peptides, and immunometabolic modulators that restore ecological balance.
5. AI-driven diagnostics combining clinical and microbiome data to stratify chronic-wound patients by risk and treatment responsiveness [103].

Ultimately, the goal is to reframe wound management from pathogen eradication toward ecological restoration, leveraging molecular understanding of host–microbiota symbiosis and powered by next-generation diagnostic technologies.

## 10. Conclusions

The skin is not a sterile barrier but a dynamic immune–metabolic ecosystem in which microbial communities orchestrate every phase of wound repair [185]. Over the past decade, advances in sequencing, metabolomics, and systems biology have revealed that the skin microbiota modulates hemostasis, inflammation, proliferation, and remodeling through molecular pathways involving TLR2/TLR4 signaling, quorum sensing, antimicrobial peptides, redox balance, and immunometabolic reprogramming [11,72,386,387].

When host–microbe equilibrium is preserved, commensal and probiotic taxa promote angiogenesis, collagen deposition, and epithelial renewal, whereas dysbiosis and biofilm formation sustain hypoxia, oxidative stress, and immune dysregulation—driving chronic, non-healing trajectories [388]. These insights underpin a paradigm shift from purely antimicrobial approaches toward microbiome-informed wound care, in which ecological modulation complements infection control.

Emerging strategies—such as topical or biomaterial-delivered probiotics and postbiotics, engineered antimicrobial peptides, quorum-sensing inhibitors, bacteriophage-based therapies, and omics-guided diagnostics—show promise in preclinical and early clinical studies. Translation to clinical practice will require standardized evidence of efficacy, safety, and cost-effectiveness.

Progress also depends on harmonized sampling and analytical workflows, adequately powered longitudinal studies, and adaptive regulatory frameworks for live biotherapeutic and microbiome-derived products [389]. The recent U.S. FDA guidance on Clinical Study Considerations for Live Biotherapeutic Products (2023) further defines expectations for early-phase trials, yet analytical and safety-validation gaps persist. Establishing harmonized data standards and safety assessment frameworks will be crucial for clinical translation.

In sum, integrating microbiome science into wound management reframes healing as a host–microbe partnership. Deepening molecular understanding of this interplay will enable precision, eco-immunological interventions that transform chronic wounds into regenerative, resilient ecosystems.

## Figures and Tables

**Table 1:** **The Impact of Different Bacteria on Wound Healing.** The table shows how different types of bacteria influence wound healing in either a positive (promoting wound healing) or negative (delaying wound healing) way.

Bacteria	Type of Study	Influence on Wound Healing	Mechanism/Host Pathway	Reference
*Staphylococcus epidermidis*	*In vivo*	Promotes wound healing	Dendritic cell–mediated CD8⁺ T cell IL-17A induction → enhanced barrier defence	[202,203]
	*In vivo* (rat incision wound model)	Promote wound healing	Topical gel application → enhanced epithelial migration and reduced inflammation	[204]
	*Ex vivo* human skin explant model (diabetic & non-diabetic)	Promote wound healing	Keratinocyte proliferation & migration ↑ → accelerated epithelial tongue length in wounded skin	[205]
*Staphylococcus hominis*	Clinical trial (human topical application)	Promote wound healing	Topical application → increased abundance on skin → enhanced barrier parameters and skin physiology	[206]
	*In vitro* and *in vivo* (mouse dermonecrosis model)		Secretion of AIPs→ agr quorum-sensing inhibition in *S. aureus* → reduced α-toxin and protease expression → attenuated tissue injury and enhanced barrier protection	[207]
*Staphylococcus aureus*	*In vivo* and *in vitro*	Delays wound healing	Activates NF-κB/MAPK signaling → upregulates connexin-43 in keratinocytes → impairs keratinocyte migration and re-epithelialization	[208]
	*In vivo* (rabbit ear wound model)		Mature biofilm formation → sustained low-grade inflammation → impaired tissue repair	[209]
	*In vitro*		Biofilm-conditioned medium → keratinocyte dendrite-like morphology & increased apoptosis → delayed scratch-closure	[210]
*Streptococcus pyogenes*	Clinical case report	Delay wound healing	Invasive *S. pyogenes* infection → skin erosion, abscess formation, and milk fistula → impaired epithelial repair and inflammation	[211]
*Pseudomonas aeruginosa*	*In vivo* (murine excisional wound model)	Delays wound healing	Secreted proteases pseudolysin (LasB) and protease IV degrade ECM proteins (fibronectin, elastin, collagen) and disrupt keratinocyte migration → suppression of TGF-β and VEGF signaling	[97]
			Reduced TNF-α, IL-1β, and IL-6 expression → attenuated macrophage recruitment and delayed fibroblast activation	[212]
*Enterococcus faecalis*	*In vivo*	Delays wound healing	*E. faecalis* formed biofilm-like microcolonies within wounds → activated TLR2–NF-κB pathway → sustained neutrophil and macrophage infiltration → chronic inflammatory microenvironment	[213]
			*E. faecalis* persisted within wound tissue → modulated innate immune activation → suppressed macrophage phagocytic function and cytokine signaling (IL-1β, TNF-α) → prolonged inflammatory phase	[214]
*Escherichia coli*	Clinical isolates from skin & soft-tissue infections (human)	Delay wound healing	Virulence genes (*cnf1*, *hlyA*, *ompT*) → tissue invasion and immune evasion	[215]
*Lactobacillus plantarum*	*In vivo* and *in vitro*	Promotes wound healing	AGEs → NLRP3 inflammasome/Caspase-1/GSDMD activation → IL-1β & IL-18 ↑ → pyroptosis; L. plantarum ↓ NLRP3 → ↓ pyroptosis → improved repair	[216]
*Lactobacillus bulgaricus* & *Lactobacillus plantarum*	*In vivo*	Promote wound healing	↓ pro-inflammatory cytokines (IL-1β, TNF-α) → reduced inflammatory cell infiltration → enhanced fibroblast migration & matrix deposition	[217]
*Lactobacillus plantarum* (*GMNL-6*) & *L. paracasei* (*GMNL-653*)	*In vitro* and *In vivo*	Promote wound healing	↑ MMP-1 early phase → ECM remodelling; ↓ α-SMA/fibrosis in later phase; lipoteichoic acid mimics effect → TGF-β/Smad2 inhibition → reduced fibrosis, faster repair	[218]

## Data Availability

This review does not involve any created of analyzed data; therefore data sharing is not appliable.
